# Accessory right hepatic artery branched from gastroduodenal artery

**DOI:** 10.1186/s40792-015-0092-7

**Published:** 2015-09-28

**Authors:** Kohei Yamashita, Daisuke Hashimoto, Rumi Itoyama, Hirohisa Okabe, Akira Chikamoto, Toru Beppu, Hideo Baba

**Affiliations:** Department of Gastroenterological Surgery, Kumamoto University Graduate School of Medical Sciences, 1-1-1 Honjo, Chuo-ku, Kumamoto City, 860-8556 Japan

**Keywords:** Vascular anomaly, Hepatic artery, Gallbladder cancer

## Abstract

The right hepatic artery usually branches from the common hepatic artery, however, there are cases showing anatomic variations. We present 41-year-old female patient with gallbladder cancer. In this case, the accessory right hepatic artery branched from the gastroduodenal artery, passed in front of the common bile duct and fed into the anterior segment of the liver. Cholecystectomy and resection of the extrahepatic bile duct with hepaticoenterostomy were performed successfully, preserving the accessory right hepatic artery. There are few reports presenting such an extremely rare anomaly of hepatic arteries in the English literature. Additionally, we herein present a review of the English literature regarding anatomic variations of right hepatic artery.

## Background

The patterns of the arterial blood supply to the liver have a tendency to show a certain variability [1, 2]. The right hepatic artery (RHA) usually arises from the common hepatic artery (CHA). One of the best known anatomic variations of hepatic arteries is a replaced or accessory RHA (aRHA) branching from the superior mesenteric artery (SMA) [3, 4]. However, we would like to present an extremely rare case of the aRHA branching from the gastroduodenal artery (GDA).

## Case presentation

**Fig. 1:**
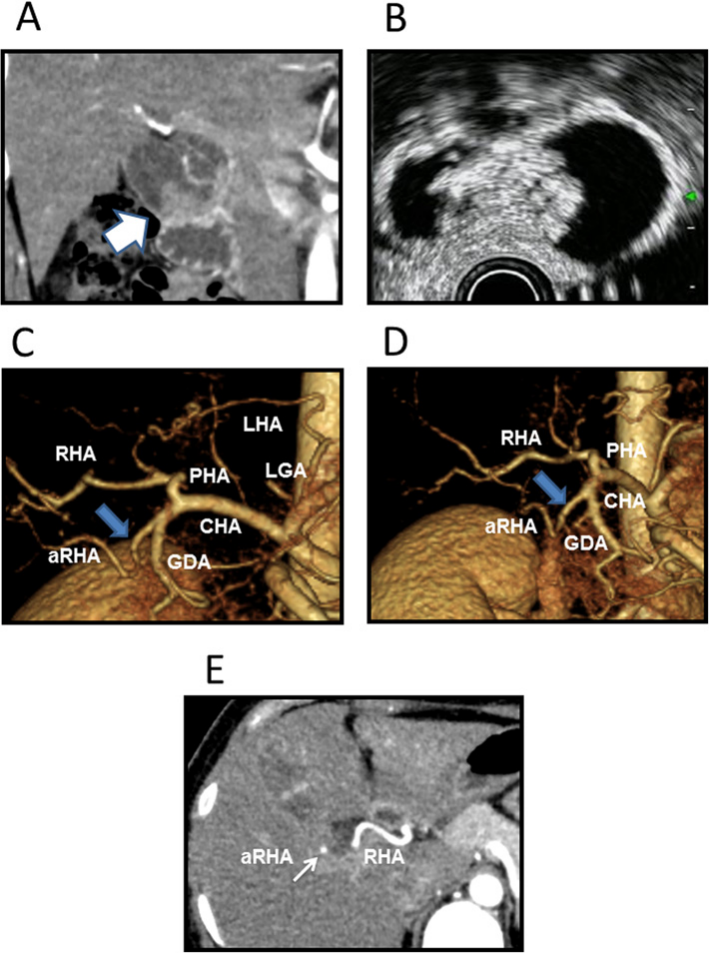
Preoperative findings. A contrast-enhanced CT (**a**) and ultrasonography (**b**) revealed a papillary hypervascular tumor, 25 × 21 mm, in the gallbladder. The 3D-CT angiography (**c**, **d**) indicated that the aRHA (*arrow*) branched from the GDA, whereas the cholecystic artery could not be detected. The aRHA (*arrow*) fed into the anterior segment of the liver (**e**). *CHA* common hepatic artery, *GDA* gastroduodenal artery, *PHA* popper hepatic artery, *aRHA* accessory right hepatic artery, *LGA* left gastric artery, *LHA* left hepatic artery

A 41-year-old woman was referred to us for gallbladder cancer. An ultrasonography and a contrast-enhanced computed tomography (CT) scan revealed a papillary hypervascular tumor, 25 × 21 mm, in the gallbladder (Fig. 1a, b). Three-dimensional (3D)-CT angiography showed that the aRHA branched from the GDA, whereas the cholecystic artery could not be detected (Fig. 1c, d). The aRHA passed in front of the common bile duct and fed into the anterior segment of the liver (Fig. 1e). The proper hepatic artery (PHA) was divided distally into the RHA and the middle hepatic artery (MHA). The left hepatic artery (LHA) was replaced on the left gastric artery (LGA). Pancreaticobiliary maljunction was not detected in this case.

**Fig. 2:**
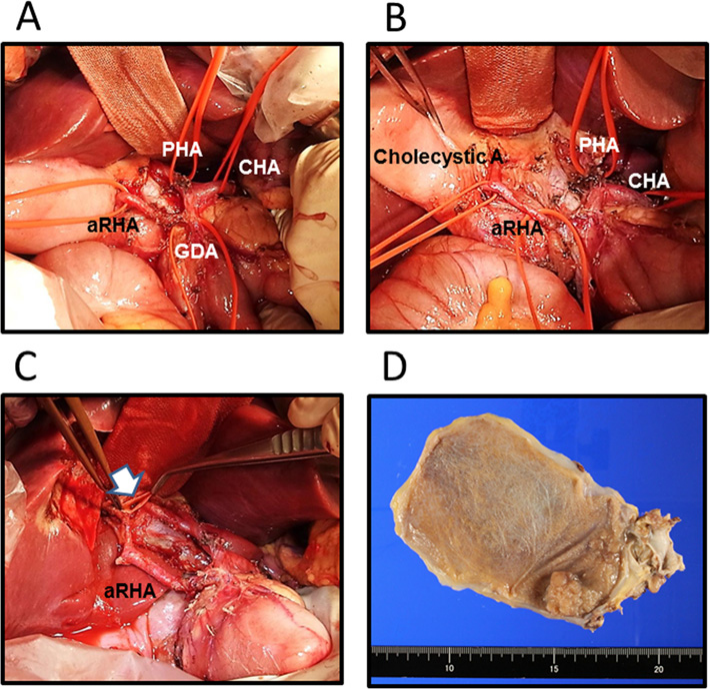
Operative findings. The aRHA branching from the GDA was detected (**a**). The cholecystic artery was found, arising from the aRHA (**b**). The gallbladder and the extrahepatic bile duct were resected, preserving the aRHA (**c**). Macroscopically, the tumor was 2.5 × 2.0 cm (**d**). *CHA* common hepatic artery, *GDA* gastroduodenal artery, *PHA* popper hepatic artery, *aRHA* accessory right hepatic artery, *LGA* left gastric artery, *LHA* left hepatic artery. *Arrow*: cut end of the bile duct

The patient underwent operation, and laparotomy revealed that there was no invasion into the liver. The aRHA branching from the GDA was detected being consistent with the preoperative 3D-CT (Fig. 2a). The cholecystic artery was found, arising from the aRHA (Fig. 2b). Finally, the cholecystic artery was cut and resection of the gallbladder, and the extrahepatic bile duct with additional hepaticoenterostomy was performed, preserving the aRHA successfully (Fig. 2c). Whereas the effectiveness of lymphadenectomy for early-stage gallbladder cancer has been controversial [5], we performed resection of the extrahepatic bile duct for lymph node dissection. Macroscopically, the tumor was 2.5 × 2.0 cm (Fig. 2d). Postoperative pathological analysis diagnosed a papillary adenocarcinoma within the mucosal layer of the gallbladder.

Michels et al. published autopsy series about hepatic artery variants in 1966 [6], and in which, they indicated that aRHA uncommonly branches from GDA. We reviewed the English literature, in which 6588 cases were analyzed about anatomic variation of hepatic artery, including the presented case [3, 7–14] (Table 1). This study was approved by the Institutional Review Board of Kumamoto University Hospital. Among 6588 cases, 5696 cases (86.5 %) had standard anatomy. Replaced RHA and aRHA were the most commonly branched from SMA (853 cases, 12.9 %), followed by celiac axis (CA) (16 cases, 0.24 %), aorta (10 cases, 0.15 %), and CHA (6 cases, 0.09 %). Two cases had rare anomalies in which replaced RHA branched from LGA or renal artery. In addition, there were three cases (0.05 %) who had replaced RHA branched from GDA. Hogendorf et al. reported an autopsy case of aRHA branched from GDA [14]. However, to our best knowledge, the presented case is the first report in which aRHA branched from GDA was detected preoperatively.

**Table 1:** Anatomic variations of replaced and accessory right hepatic artery

Author	Total cases (*n*)	Origin of RHA or accessory RHA (*n*)											
		Standard anatomy	Replaced RHA from SMA	Accessory RHA from SMA	Replaced RHA from CA	Accessory RHA from CA	Replaced RHA from CHA	Replaced RHA from aorta	Accessory RHA from aorta	Replaced RHA from renal artery	Replaced RHA from LGA	Replaced RHA from GDA	Accessory RHA from GDA
Hiatt J. [3]	1000	871	129		0	0	0	0	0	0	0	0	0
Covey A. [7]	600	512	55	33	0	0	0	0	0	0	0	0	0
Gruttadauria S. [11]	701	572	110		5		2	8		1	1	2	0
Koops A. [8]	604	520	60	21	1	1	0	1	0	0	0	0	0
Abdullah S. [12]	932	772	155		5		0	0	0	0	0	0	0
Lopez-Andujar R. [9]	1081	946	118	17	0	0	0	0	0	0	0	0	0
Winston C. [13]	371	347	15	0	4	0	4	0	0	0	0	1	0
Loschner C. [10]	1297	1156	103	37	0	0	0	0	1	0	0	0	0
Hogendorf P. [14]	1	(–)	(–)	(–)	(–)	(–)	(–)	(–)	(–)	(–)	(–)	(–)	1
This report	1	(–)	(–)	(–)	(–)	(–)	(–)	(–)	(–)	(–)	(–)	(–)	1
Total	6588	5696	853		16		6	10		1	1	3	2

*RHA* right hepatic artery, *SMA* superior mesenteric artery, *CA* celiac axis, *CHA* common hepatic artery, *LGA* left gastric artery, *GDA* gastroduodenal artery

## Conclusion

In this case, the successful outcome of the operation was made possible by identifying the aRHA preoperatively. The aRHA should be preserved because it fed the anterior segment of the liver. In addition to the abnormal aRHA, this case had a replaced LHA which the use of the preoperative 3D-CT angiography helped to establish beforehand. We believe that this extremely rare arterial pattern should be known by surgeons.

## Consent

Written informed consent was obtained from the patient for publication of this case report and any accompanying images. A copy of the written consent is available for review by the Editor-in-Chief of this journal.

## Abbreviations

aRHA: accessory right hepatic artery
CA: celiac axis
CHA: common hepatic artery
CT: computed tomography
GDA: gastroduodenal artery
LGA: left gastric artery
LHA: left hepatic artery
MHA: middle hepatic artery
PHA: proper hepatic artery
SMA: superior mesenteric artery
